# Randomized, double-blind, placebo-controlled study to evaluate the effect of treatment with an SPMs-enriched oil on chronic pain and inflammation, functionality, and quality of life in patients with symptomatic knee osteoarthritis: GAUDI study

**DOI:** 10.1186/s12967-023-04283-4

**Published:** 2023-06-29

**Authors:** Ingrid Möller, Gil Rodas, Jose María Villalón, Jose A. Rodas, Francisco Angulo, Nina Martínez, Josep Vergés

**Affiliations:** 1 Institut Poal de Reumatología, Barcelona, Spain; 2 FC Barcelona, Barcelona, Spain; 3 FC Atlético de Madrid, Madrid, Spain; 4 Asturias Football Federation, Avilés, Spain; 5 Athletic FC, Bilbao, Spain; 6 Osteoarthritis Foundation International (OAFI), Barcelona, Spain

**Keywords:** Specialized pro-resolving mediators, SPMs, Resolvins, Supplementation, Osteoarthritis, Chronic pain, Quality of life, Placebo

## Abstract

**Background:**

Specialized pro-resolving mediators (SPMs), including 18-HEPE, 17-HDHA, and 14-HDHA are recognized as potentially therapeutic in inflammatory diseases because SPMs regulate the inflammation process, which leads to, for example; swelling and the sensation of pain. In osteoarthritis (OA), chronic pain is described as the symptom that reduces patients´ quality of life (QoL). The GAUDI study evaluated the efficacy of SPMs supplementation in reducing pain in the symptomatic knee of OA patients.

**Methods:**

This randomized, multicenter, double-blind, and placebo-controlled parallel-group pilot study was performed in Spain and conducted on adults 18–68 years old diagnosed with symptomatic knee OA. Patients were enrolled in the study for up to 24 weeks, which included a 12-week intervention period and a follow-up visit on week 24. The primary endpoint was pain change measured through a Visual Analog Scale (VAS). Secondary endpoints included: Pain change evaluation, stiffness, and function according to the WOMAC index; assessment of constant, intermittent, and total pain according to the OMERACT-OARSI score; evaluation of changes in health-related QoL parameters; the use or not of concomitant, rescue, and anti-inflammatory medication; and safety and tolerability assessments.

**Results:**

Patients were enrolled in the study from May 2018 to September 2021. VAS pain score was evaluated in the per protocol population (n = 51 patients), in which we observed a statistically significant reduction after 8 weeks (p = 0.039) and 12 weeks (p = 0.031) of treatment in patients consuming SPMs (n = 23 subjects) vs. placebo (n = 28 subjects). In line with the OMERACT-OARSI score, intermittent pain was reduced after 12 weeks with statistical significance (p = 0.019) in patients treated with SPMs (n = 23 subjects) vs. placebo (n = 28 subjects). Functional status as WOMAC score did not significantly change after SPMs or placebo consumption. Notably, patients consuming SPMs showed improvements in all five aspects of the EUROQoL-5, including a significant improvement in the usual-activities dimension. None of the patients required rescue medication, nor were any adverse events reported.

**Conclusions:**

These findings suggest that sustained SPMs consumption reduces pain in OA patients while also improving their Quality of Life. These results also support the safety profile of SPMs supplementation.

*Trial registration* NCT05633849. Registered 1 December 1 2022. Retrospectively registered, https://clinicaltrials.gov/ct2/show/study/NCT05633849

**Supplementary Information:**

The online version contains supplementary material available at 10.1186/s12967-023-04283-4.

## Background

Osteoarthritis (OA) is the most common form of arthritis and a significant cause of pain, functional disability, and socioeconomic cost worldwide. OA is a disease that involves inflammatory, mechanical, and metabolic factors. All these lead to pathological changes across different weight-bearing joint tissues, severely and particularly affecting cartilage tissue [1, 2]. Chronic pain, as the main symptom of OA, presents itself as a multifaceted pathophysiology involving central and peripheral neurological mechanisms. It can clinically manifest itself as; severe, intermittent and persistent background pain, with variable intensity and sensation such as: burning, tingling, numbness, and pins and needles [3, 4]. Thus, chronic pain negatively impacts social connectedness and psychological well-being. Overall, pain reduces OA patients´ quality of life (QoL) [5, 6].

Infiltration of immune cells and release of pro-inflammatory mediators activate different inflammatory pathways in joints. These lead to the release of pro-nociceptive molecules, which induce peripheral sensitization [1, 7]. Several mediators, consisting of prostaglandins, cytokines, neuropeptides, and proteinases, contribute to initiation and uninterrumpted joint inflammation and associated pain [7]. Under normal circumstances, pain plays a protective role [3]. However, inadequate resolution of inflammation produces persistent inflammation and chronic pain, which does not protect nor support healing and as such is considered maladaptive [8]. The resolution of inflammation is an active biochemical process mainly driven by specialized pro-resolving lipid mediators (SPMs) [9]. SPMs derive from essential polyunsaturated fatty acids (PUFAs), namely arachidonic acid (AA), eicosapentaenoic acid (EPA), and docosahexaenoic acid (DHA). PUFAs can be metabolized into potent anti-inflammatory and pro-resolving mediators with anti-nociceptive effects as monohydroxylated mediators: 18-hydroxyeicosapentaenoic acid (18-HEPE), 17-hydroxydocosahexaenoic acid (17-HDHA), 14-hydroxydocosahexaenoic acid (14-HDHA), among others. Furthermore, monohydroxylated SPMs are also metabolized into other di- and tri-hydroxylated SPM families, namely; lipoxins, resolvins, protectins, and maresins [10]. Bioactive concentrations of SPMs have been established in human organs, tissues, and fluids of all kinds, including plasma, and serum [11], bone marrow [12], placenta [13], synovial fluid [14], lymph nodes [15], spleen [15, 16], adipose tissue [17], breast milk [18], urine [19], vagus nerve [20], cerebral spinal fluid [21], and the brain [22, 23].

At the cellular level, SPMs act via specific receptors that promote macrophage phagocytosis of: pathogens, apoptotic cells, and cellular debris, that precede and is necessary for tissue regeneration. SPMs also cease infiltration of pro-inflammatory immune cells, counter-regulate pro-inflammatory mediators, and increase the production of anti-inflammatory mediators [24]. Resolvins, for example, have been shown to regulate pain in neuropathic and inflammatory animal models [25]. SPMs carry out their actions through GPCR receptors, playing critical roles in cell signaling [26–28]. With remarkable precision, SPMs limit the infiltration of neutrophils to the inflammatory focus, promoting the clearance of apoptotic cells and cellular debris. Their power to enhance efferocytosis, counter-regulate the production of pro-inflammatory mediators like chemokines and cytokines, and promote pro-resolving macrophage skewing is of paramount importance. SPMs guide the adaptive immune system and instigate tissue repairment and regeneration processes [29, 30]. Thus, SPMs range of action spans the mammalian body, touching upon afflictions such as arthritis [29], sepsis [27], diabetes, atherosclerosis [31], Alzheimer's disease [32], and inflammatory bowel disease, among others [33].

Despite OA´s prevalence and burden, its standard treatment still involves the use of non-steroidal anti-inflammatory drugs (NSAIDs), which lack sufficient efficacy and present multiple side effects. With limited pharmacological options, in many cases joint replacement is considered as the only treatment option [2, 3, 7, 34]. Recently, SPMs administered orally to healthy individuals, peripheral artery disease patients, and obese patients have been shown to: Increase SPMs levels in peripheral blood, activate downstream lipid mediator pathways, dampen inflammation, and induce a more pro-resolution phenotype in circulating leukocytes and macrophages [35–37]. There is also clinical evidence showing the efficacy of 17-HDHA and 18-HEPE in improving QoL and reducing pain in adults with chronic pain [38].

In this study, the effect of consuming an SPMs enriched oil has been clinically evaluated in the context of pain in patients with OA. This randomized, double-blind, placebo-controlled trial sought to assess the efficacy of SPMs 12-week consumption in reducing pain in patients with symptomatic knee OA.

## Methods

### Study design

The GAUDI study was a randomized, multicenter, double-blind, placebo-controlled, parallel-group pilot study conducted in 5 Spanish centers in compliance with the World Medical Association Declaration of Helsinki, all its amendments, and national regulations. The Independent Ethic Committee of Hospital Universitario La Paz (Madrid, Spain) approved this study. All patients gave their written informed consent.

**Fig. 1:**
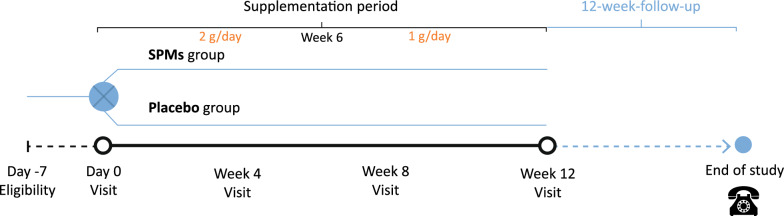
GAUDI Study design diagram

The study duration per patient was up to 24 weeks consisting of a screening period, a treatment period with monthly visits from the start of the study until week 12, and a last follow-up visit on week 24 conducted by telephone call (Fig. 1).

For the treatment period, participants were instructed to take two 500 mg softgels of SPMs enriched oil (SPMs group) or olive oil placebo (placebo group) after breakfast and two 500 mg softgels after dinner for a total of four softgels of SPMs or placebo per day during the first 6 weeks of the study. During the last 6 weeks, participants were instructed to take one softgel after breakfast and one after dinner for a total of two softgels of SPMs or placebo per day. Thus, the treatment period lasted in total 12 weeks per patient.

### Patient population

Eligible patients for the study were adults between 18 and 68 years old that were diagnosed with symptomatic knee OA (according to the American College of Rheumatology [ACR]) and primary knee OA with both confirmed scores of 2–3 on the Kellgren and Lawrence radiological scale [39] and 5 or more on the pain Visual Analogue Scale (VAS). Patients were excluded from the study if they were allergic to fish or seafood, had an arthroscopy within the last year, or had been diagnosed with secondary knee OA, cardiopathy, pneumopathy, non-compensated nephropathy, neuropathy affecting mobility, non-compensated psychiatric disorder, fibromyalgia, and/or cognitive disorder. Patients were also excluded if they had received chondroitin sulfate, glucosamine, diacerein, corticoids infiltration, and/or platelet-rich plasma infiltration in the knee 3 months prior to inclusion, NSAIDs 3 weeks before inclusion, and/or hyaluronic acid infiltration in the knee 6 months prior to inclusion in the study. Patients could not have had a drug abuse record for 3 years before the inclusion in the study nor have been participating in any another clinical trial at the same time.

### Study endpoints

The primary endpoint was the change in pain measured as VAS score before supplementation and VAS score on week 12 of supplementation. Secondary endpoints included the comparison in pain change, stiffness, and joint function according to the Western Ontario and McMaster Universities Osteoarthritis Index (WOMAC) between SPMs and placebo groups before supplementation to week 12. In addition, the assessment of constant, intermittent, and total pain according to the OMERACT-OARSI score was considered. Changes in health-related QoL scores measured by the EUROQoL-5 questionnaire [40] between placebo and SPMs groups were also assessed. Finally, the use of concomitant, rescue, and anti-inflammatory medication and the incidence of adverse events (AEs) were considered.

### Statistical considerations

Three patient populations were considered in analyses: (i) The per protocol (PP) population, including all randomized patients who received at least one treatment dose and had all primary endpoint measurements and no major protocol deviations; (ii) the intention-to-treat (ITT) population, including all randomized patients; (iii) and the safety population, with all randomized patients who received at least one dose of the study treatment. The PP population dataset was the only population considered for the primary analysis.

For univariate analysis, the quantitative variables were described using central tendency and dispersion measures, including mean, standard deviation (SD), median and interquartile range (IQR). To define qualitative variables, total counts and percentages were used.

For bivariate analysis between distinct subjects, quantitative variables were analyzed using the Student's t-test for independent samples or the Mann–Whitney U test when samples were not normally distributed. Qualitative variables were analyzed using the Chi-square test or the Fisher test. For intra-subject variables, quantitative variables were analyzed using the Student's t-test or the Wilcoxon signed-rank test, depending on whether the samples were or were not normally distributed.

Missing data were discarded in the analyses, and a significance level of 0.05 was used in statistical testing. All statistical analyses were performed using the Statistical Package for the Social Sciences (SPSS) version 22.0 (SPSS Inc, Chicago, USA).

## Results

### Patient characteristics

One hundred patients were recruited in the GAUDI study from May 2018 to September 2021. Fifteen enrolled patients were excluded from the study because they did not fulfill the selection criteria. Therefore, 85 patients were included in the analyses and constituted the ITT population. The PP and safety populations comprised 51 and 52 patients, respectively. In the PP population, 23 patients were assigned to the SPMs group and 28 to the placebo group.

Patients characteristics upon inclusion are presented on Table 1 and Additional file 1: Table S1. Patients´s median age was 61.2 years old in the SPMs group and 57.3 years old in the placebo group. Regarding gender distribution, 52.17% of patients were female in the SPMs group, and 53.57% were female in the placebo group. Mean body mass index (BMI), systolic blood pressure (SBP), diastolic blood pressure (DBP), and heart rate were not significantly different between groups. Before treatment started, a medical history of arterial hypertension was more frequent in the SPMs group than in the placebo group (39.13% and 17.86%, respectively; p-value = 0.039). Smoking, drinking, nutrition, and physical activity habits did not significantly vary between the patients consuming SPMs or placebo. Most patients had mild (grade 2) OA according to the Kellgren and Lawrence classification: 73.91% in the SPMs group and 67.86% in the placebo group. Following the ACR criteria, quantification of knee pain and osteophytosis were 78.26% in the SPMs group and 78.57% in the placebo group.

**Table 1:** Baseline patient characteristics (N = 51)

Patient characteristics	Values		P-value
	SPMs (N = 23)	Placebo (N = 28)	
Median age, years (IQR)	61.2 (54.5–64.6)	57.3 (52.7–61.5)	0.512 ^(1)^
Female, n (%)	12 (52.17)	15 (53.57)	0.921 ^(2)^
Ethnicity, n (%):			
Caucasian	22 (95.65)	28 (100.00)	0.451 ^(3)^
Other	1 (4.35)	0 (0.00)	
*Anthropometric data*			
Mean weight, kg (SD)	76.7 (13.98)	82.5 (23.17)	0.291 ^(4)^
Mean body mass index, kg/cm^2^ (SD)	27.9 (4.44)	29.8 (10.19)	0.418 ^(4)^
*Vital signs*			
Mean SBP (mmHg) (SD)	129.4 (15.05)	128.5 (14.74)	0.399 ^(4)^
Mean DBP (mmHg) (SD)	82.3 (10.09)	78 (11.93)	0.058 ^(4)^
Mean heart rate (bpm)(SD)	70.6 (6.72)	69.9 (9.43)	0.764 ^(1)^
*Toxic habits/lifestyle*			
Smoking habits			0.220 ^(3)^
Current smoker, n (%)	2 (28.57)	1 (16.67)	
Ex-smoker, n (%)	4 (57.14)	5 (83.33)	
Alcohol drinker, n (%)	7 (100.00)	6 (100.00)	
Feeding habits			
Fish consumption, n (%):	23 (100.00)	27 (96.43)	0.549 ^(3)^
Nut consumptions, n (%):	20 (86.96)	24 (85.71)	0.313 ^(3)^
Physical activity habits			
Exercise, n (%)	22 (95.65)	23 (82.14)	0.126 ^(3)^
Mean minutes per week (SD)	192.3 (115.67)	279.6 (175.74)	0.057 ^(1)^
Exercise Intensity			0.075 ^(3)^
Low	14 (63.64)	12 (52.17)	
Moderate	7 (31.82)	11 (47.83)	
High	1 (4.55)	0 (0.00)	
*Medical history*			
Arterial hypertension	9 (39.13)	5 (17.86)	0.039 ^(3)^
Chondromalacia	5 (21.74)	6 (21.43)	
Rheumatoid arthritis	1 (4.35)	1 (3.57)	
Other	3 (13.04)	2 (7.14)	
*Osteoarthritis-related data*			
Kellgren y Lawrence classification, n (%)			0.637 ^(2)^
Mild (Grade 2)	17 (73.91)	19 (67.86)	
Moderate (Grade 3)	6 (26.09)	9 (32.14)	
ACR Criterium, n (%) ^(5)^			0.086 ^(3)^
I	18 (78.26)	22 (78.57)	
II	3 (13.04)	2 (7.14)	
III	2 (8.70)	4 (14.29)	
Osteoarthritis family history, n (%)	15 (65.22)	16 (57.14)	0.557 ^(2)^
Family history of rheumatoid disease, n (%)	0 (0.00)	1 (3.57)	0.549 ^(3)^

^**(1)**^ Student’s t-test

^**(2)**^ Chi square test

^**(3)**^ Fisher test

^**(4)**^ Mann–Whitney U test

^**(5)**^ I: knee pain and osteophytosis; II: knee pain, synovial fluid signs of OA (clear, viscous, or white blood cell count < 2000/mL), crepitus, and morning stiffness ≤ 30 min; III: knee pain, age ≥ 40 years, crepitus, and morning stiffness ≤ 30 min

ACR: American College of Rheumatology; bpm: beats each minute; DBP: diastolic blood pressure; IQR: interquartile range; SBP: systolic blood pressure; SD: standard deviation; SPMs: specialized pro-resolving lipid mediators

### Pain changes in OA patients treated with SPMs

**Fig. 2:**
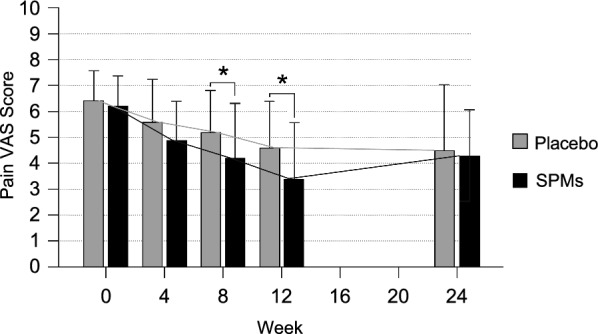
Pain in OA patients according to VAS (N = 51). Means (standard deviation) are used. To compare the VAS Scores between the SPMs group (N = 23) and placebo group (N = 28), the Mann–Whitney U test (basal) and the Student's t-test (weeks 4, 8, and 12) were used. * p-value < 0.05. SPMs: specialized pro-resolving lipid mediators; VAS: Visual Analogue Scale

There was a statistically significant lower pain VAS score in the SPMs group compared to the placebo group after 8 weeks (4.2 ± 2.11 and 5.2 ± 1.6, respectively; p-value = 0.039) and 12 weeks (3.4 ± 2.17 and 4.6 ± 1.79; p-value = 0.031) of treatment (Fig. 2). In addition, a trend of higher reduction in pain VAS score changes was observed from baseline to week 12 in the SPMs group compared to the placebo group (reduced by 44.8 ± 37.44% vs. reduced by 28.4 ± 24.44%, respectively; p-value: 0.066).

To assess SPMs long-lasting residual effects, pain VAS score was evaluated on week 24 (12 weeks after treatment completion). From weeks 12–24, pain VAS score changes increased by 50.1 ± 115.60% in the SPMs group and 2.5 ± 65.69% in the placebo group, respectively (p-value = 0.105). On week 24, the pain VAS score was similar between placebo and SPMs groups (4.5 ± 2.53 and 4.3 ± 1.77, respectively; p-value = 0.372).

Constant, intermittent, and total pain, as measured with the OMERACT-OARSI method, did not significantly vary between patients receiving SPMs or placebo at any time. The intermittent pain changes significantly decreased by: 30.6 ± 29.30% in the SPMs group and by 18.1 ± 25.53% in the placebo group (p-value = 0.019) (Table 2). After 12 weeks of supplementation, there was a tendency to larger changes in total pain in the SPMs group (decreased by 30.7 ± 28.26%) compared to the placebo group (decreased by 20.7 ± 26.15%; p-value = 0.091).

**Table 2:** Pain changes in OA patients according to OMERACT-OARSI (N = 51)

OMERACT-OARSI ^(1)^	Values		P-value
	SPMs (N = 23)	Placebo (N = 28)	
Constant pain			
Mean change from baseline to week 12, % (SD)	− 30.0 (29.10)	− 20.8 (35.48)	0.320 ^(2)^
Intermittent pain			
Mean change from baseline to week 12, % (SD)	− 30.6 (29.30)	− 18.1 (25.53)	0.019 ^(3)^
Total pain			
Mean change from baseline to week 12, % (SD)	− 30.7 (28.26)	− 20.7 (26.15)	0.091 ^(3)^

Percentage change in each patient has been used to calculate the mean score change

^(1)^ Constant, intermittent, and total scores resulted from the summation of the scores of the individual item answers; changes are shown in percentages; ^(2)^ Student's t-test; ^(3)^ Mann -Whitney U test

OMERACT-OARSI: Standing Committee for Clinical Trials Response Criteria Initiative and the Outcome Measures in Rheumatology-Osteoarthritis Research Society International; SD: standard deviation; SPMs: specialized pro-resolving lipid mediators

### Functional changes in OA patients treated with SPMs

**Fig. 3:**
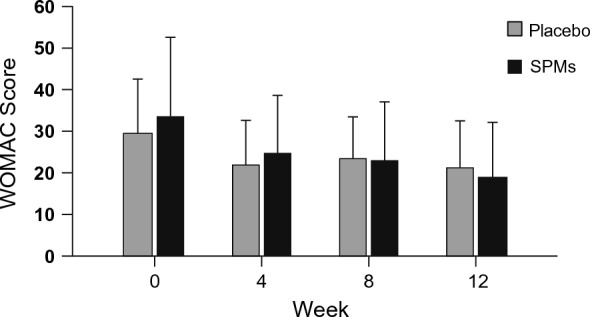
Functional status of OA patients according to WOMAC (N = 51). Means (standard deviation) are used. Total score derived from the summation of the scores of the answers of the 24 items, which correspond to: none: 0, mild: 1, moderate: 2, severe: 3, and extreme: 4. To compare the WOMAC scores between the SPMs group (N = 23) and placebo group (N = 28), the Mann–Whitney U test (basal) and the Student's t-test (weeks 4, 8, and 12) were used. SPMs: specialized pro-resolving lipid mediators; WOMAC: Western Ontario and McMaster Universities Osteoarthritis Index

The WOMAC score mean did not significantly differ between patients in the SPMs and the placebo groups at any recorded time (at t = 0: SPMs group: 33.6 ± 19.04, placebo group: 29.6 ± 12.97, p-value = 0.377; at week 4: SPMs group: 24.9 ± 13.71, placebo group: 22.0 ± 10.62, p-value = 0.391; at week 8: SPMs group: 23.1 ± 13.96, placebo group: 23.5 ± 9.97, p-value = 0.921; at week 12: SPMs group: 19.2 ± 12.98, placebo group: 21.3 ± 11.23, p-value = 0.529) (Fig. 3). Of note, patients receiving SPMs tended to show a larger reduction in the WOMAC score from before treatment to week 12 (reduced by 41.4 ± 36.30%) than patients in the placebo group (reduced by 18.0 ± 54.35%; p-value: 0.082).

### Quality of life

Overall, patients in the SPMs group showed improvements in all five dimensions of the EUROQoL-5 compared to the placebo group, including significant changes in the usual activities dimension (52.17% vs. 14.29%; p-value = 0.004) (Table 3). Patients in the SPMs group also showed trends towards improvement in mobility (43.48% vs. 25.00%; p-value = 0.164), self-care (30.43% vs. 17.86%; p-value = 0.292), pain/discomfort (60.87% vs. 46.43%; p-value = 0.304), and anxiety/depression (26.09% vs. 14.29%; p-value = 0.162).

**Table 3:** Quality of life in OA patients (N = 51)

Parameter	Values		P-value
	SPMs (N = 23)	Placebo (N = 28)	
EQ VAS			
Mean change from baseline to week 12, % (SD)	51.8 (216.71)	48.6 (204.17) ^(1)^	0.404 ^(2)^
Mobility dimension			
Improvement, n (%)	10 (43.48)	7 (25.00)	0.164 ^(3)^
Self-care dimension			
Improvement, n (%)	7 (30.43)	5 (17.86)	0.292 ^(3)^
Usual activities dimension			
Improvement, n (%)	12 (52.17)	4 (14.29)	0.004 ^(3)^
Pain/discomfort dimension			
Improvement, n (%)	14 (60.87)	13 (46.43)	0.304 ^(3)^
Anxiety/depression dimension			
Improvement, n (%)	6 (26.09)	4 (14.29)	0.162 ^(4)^

Percentage change in each patient has been used to calculate the mean score change. ^(4)^ SPM: N = 23, Placebo: N = 27); ^(2)^ Mann–Whitney U test; ^(3)^ Chi-Square test; ^(4)^ Fisher’s test; (4) missing data: Placebo, N = 1

EQ VAS: EuroQol Visual Analogue Scale; SPMs: specialized pro-resolving lipid mediators

### Concomitant, rescue, and anti-inflammatory medication

None of the patients required rescue medication (Table 4). Before treatment, 10 (43.48%) patients in the SPMs group and seven (25%) patients in the placebo group were using concomitant medication (p-value = 0.164), while three (13.04%) patients in the SPMs group and four (14.29%) patients in the placebo group were using concomitant medication on week 12 (p-value = 0.313). After the treatment period and until week 24, four patients in each group (17.39% in the SPMs group and 14.29% in the placebo, p-value = 0.285) received anti-inflammatory medication.

**Table 4:** Concomitant, rescue, and anti-inflammatory medication (N = 51)

Medication	Values		P-value
	SPMs (N = 23)	Placebo (N = 28)	
Concomitant medication at baseline, n (%)			
At baseline	10 (43.48)	7 (25.00)	0.164 ^(1)^
At week 4	2 (8.70)	2 (7.14)	0.383 ^(2)^
At week 8	2 (8.70)	2 (7.14)	0.383 ^(2)^
At week 12	3 (13.04)	4 (14.29)	0.313 ^(2)^
Rescue medication, n (%)			
At week 4	0 (0.00)	0 (0.00)	
At week 8	0 (0.00)	0 (0.00)	
At week 12	0 (0.00)	0 (0.00)	
Anti-inflammatory medication, n (%)			
At week 24	4 (17.39)	4 (14.29)	0.285 ^(2)^

^(1)^ Chi-Square test; ^(2)^ Fisher's test. SPMs: specialized pro-resolving lipid mediators

### Safety and tolerability

Only two AEs were reported in one patient from the SPMs group (lameness and locked knee), which were mild and not related to the study treatment. These did not require any action from the research team and were resolved. None of the patients withdrew from the study.

## Discussion

The results of this randomized, double-blind, placebo-controlled study imply that oral supplementation with SPMs reduces pain and improves QoL in patients with symptomatic knee OA. While a placebo effect is observed in clinical variables, this is not as significant as the effect of SPMs consumption. Following a dose reduction from 2000 mg/day to 1000 mg/day from weeks 6 to 12, patients receiving oral supplementation with SPMs reported a statistically significant reduction in pain after 8 and 12 weeks of treatment compared to patients in the placebo group. Also, we observed a trend, such as the reduction in the pain VAS score up to week 12 and changes in VAS score from weeks 12 to 24, that added to the impact of continuous SPMs consumption. Altogether, these results support the recommended dual dose for OA patients.

OA patients identify two types of pain: constant background pain and intermittent severe pain [41]. The latter typically results in reduced physical functioning and tends to significantly impact patients' quality of life in their daily activities, social interactions, mood, and sleep [42, 43]. In our study, and according to the OMERACT-OARSI score, intermittent pain improved in patients consuming SPMs. Additionally, and as defined by EUROQoL-5 as usual activities, mobility, self-care, pain/discomfort, and anxiety/depression, there was a significant improvement in the usual activities variable with a general tendency for improvement in the health state of patients that consumed SPMs. In addition, according to WOMAC, higher functional improvement was observed in patients receiving SPMs, despite displaying higher baseline levels than placebo patients. Taken together, results from three independent methods, OMERACT-OARSI, WOMAC, and EUROQoL-5, indicate that SPMs supplementation reduces pain in OA patients.

Pain reduction associated with SPMs administration is in line with previous studies pertaining to inflammation resolution mechanisms. Preclinical data from different animal models of osteoarthritis, acute inflammatory pain, and chronic adjuvant-induced arthritis support the pain-relieving properties of 17-HDHA and resolvin D1 exogenous administration [44, 45]. Notably, the impact of SPMs on analgesic mechanisms in vitro and in vivo has been previously demonstrated. A study in rats reported on the effectiveness of resolvin D1 (RvD1) in reducing acute and chronic postoperative pain. Injection of RvD1 before surgery significantly reduced primary mechanical hypersensitivity and overall pain levels during the 10-day postoperative period. Delaying the injection of RvD1 resulted in incomplete pain reversal, suggesting that resolvins are more effective in the early stages of postoperative pain [46]. Another study in rats highlighted the potential of intrathecal resolvins (including RvD1 and RvD2) in preventing chronic post-thoracotomy pain by reducing mechanical hypersensitivity and the occurrence of vigorous nocifensive responses [47]. In humans, 17-HDHA in blood, but not resolvins D1, D2, D3, D5, and E1, have been connected to lowering pain scores in OA patients [48]. Although there is still a limited number of studies focused on the beneficial effects of SPMs supplementation in humans, early evidence of the efficacy of SPMs consumption has been shown in subjects with chronic pain [38].

To our knowledge, this is the first-in-human study evaluating possible residual effects of SPMs months after treatment. Our results do not support a long-lasting residual effect of SPMs consumption on chronic pain 12 weeks after the completion of treatment, suggesting the need for continuous supplementation to maintain clinical benefits. Preclinical studies, however, have evaluated the long-lasting effects of SPMs. A study in surgical pain in a mouse model demonstrated that intrathecal-injected resolvin D1 showed an analgesic effect for up to 30 days [46]. Another study using a mouse model of acute inflammatory pain showed a long-lasting analgesic effect of Maresin 1 (only up to 5 days) [49]. While these animal model-based studies focused on acute pain, these might not extrapolate to human chronic pain. In addition, 12 weeks after treatment may be excessively long to study residual effects. Thus, further studies will be needed to fully understand the long-term residual effect (i.e. 1–10 weeks after treatment) with SPMs supplementation in humans.

Essentially, chronic pain involves central and peripheral neurological mechanisms. Preclinical studies have shown that SPMs administration dampens inflammatory pain [50]. In addition, mechanistic studies in animal models of OA have suggested that the predominant mechanism of action involves resolvins [44]. Whether SPMs exert their function in OA joints and/or in the central nervous system in humans would require additional investigation.

In this study, SPMs supplementation did not change concomitant, rescue, and anti-inflammatory medication use. Also, no adverse events related to supplementation nor any tolerability issues were reported in this study. These data support the favorable safety record of SPMs supplementation with the administered dose, which agrees with previous studies [35].

Some limitations of this study included the modest sample size, which might have constrained the statistical significance of the results and masked the full clinical potential of SPMs consumption by OA patients. Despite the low number of participants, there is a general trend of improvement in most of the assessed clinical parameters, supporting the potential benefits to be confirmed in a larger sample number. Another limitation is the use of olive oil softgels as placebo. Olive oil has been reported to have clinical effects on OA patients, as previously reported in randomized controlled trials [51, 52]. All clinical parameters evaluated in this study showed a pattern of improvement in OA patients in the placebo group, which could be attributed to the placebo effect and/or the olive oil effect.

One future study will involve the analysis of the biochemical parameters from plasma and serum samples of the patients on this study, including SPMs and cytokines. Other studies could be performed on assessing pain reduction in patient subpopulations. For example, studying the effect of SPMs consumption in patients with one specific degree of osteoarthritis according to the Kellgren and Lawrence classification. Patients with other diseases that also experience chronic pain may benefit from SPMs consumption, though; studies that support that need to be rigorously performed. As individual SPMs are made available in their purest forms, future studies could be performed to treat joint degeneration locally, instead of systemically.

## Conclusions

This randomized, double-blind, placebo-controlled study evaluates for the first time the effect of continuous oral SPMs consumption on an adult population of symptomatic knee OA patients. SPMs supplementation reduced pain and improved the quality of life of OA patients. Our results do not support a long-lasting residual effect of SPMs after treatment, suggesting the need for continuous SPMs supplementation to maintain some of the clinical benefits. The results from this study support the favorable safety record of SPMs-enriched oil consumption.

## Supplementary Information

**Additional file 1: Table S1.** Blood test results (N = 51).

## Data Availability

The datasets analyzed during the current study are available from the corresponding author upon reasonable request.

## Abbreviations

14-HDHA: 14-Hydroxydocosahexaenoic acid
17-HDHA: 17-Hydroxydocosahexaenoic acid
18-HEPE: 18-Hydroxyeicosapentaenoic acid
AA: Arachidonic acid
ACR: American College of Rheumatology
AEs: Adverse events
BMI: Body mass index
DBP: Diastolic blood pressure
DHA: Docosahexaenoic acid
EPA: Eicosapentaenoic acid
IQR: Interquartile range
ITT: Intention-to-treat
NSAIDs: Non-steroidal anti-inflammatory drugs
OA: Osteoarthritis
PP: Per protocol
PUFAs: Polyunsaturated fatty acids
SBP: Systolic blood pressure
SD: Standard deviation
SPMs: Specialized, pro-resolving mediators
SPSS: Statistical package for the social sciences
VAS: Visual analogue scale
WOMAC: Western ontario and mcmaster universities osteoarthritis index
