# Supplementary material for: Randomized, double-blind, placebo-controlled study to evaluate the effect of treatment with an SPMs-enriched oil on chronic pain and inflammation, functionality, and quality of life in patients with symptomatic knee osteoarthritis: GAUDI study
Source: J Transl Med. 2023 Jun 29;21:423. doi: 10.1186/s12967-023-04283-4 (PMC10308764; doi:10.1186/s12967-023-04283-4)
Supplement: Supplementary file 1 — Additional file 1: Table S1. Blood test results (N = 51). [file 12967_2023_4283_MOESM1_ESM.docx]

Additional Tables

Additional Table 1. Blood test results (N = 51)

| **Variable** | **Baseline** | | | | **Week 12** | | | |
| --- | --- | --- | --- | --- | --- | --- | --- | --- |
|  | **SPMs** | **Placebo** | **P-value ^(1)^** | **N** | **SPMs** | **Placebo** | **P-value ^(1)^** | **N** |
| **Hemoglobin**, g/dL | 15 (0.84) | 14.9 (1.15) | 0.796 | 18 | 15.9 (1.01) | 13.8 (1.11) | 0.05 | 7 |
| **Hematocrit**, L/L | 44.4 (2.18) | 45.2 (2.87) | 0.501 | 18 | 47.5 (3.12) | 41.9 (3.31) | 0.073 | 7 |
| **Platelet**, 10/L | 183.3 (27.91) | 187.7 (80.91) | 0.884 | 18 | 212.7 (27.15) | 183.8 (110.09) | 0.682 | 7 |
| **PTT**, seconds | 55.6 (35.71) | 65 (36.90) | 0.145 ^(2)^ | 18 | 74.7 (43.88) | 64.5 (40.99) | 0.765 | 7 |
| **TT**, seconds | 71.5 (36.99) | 63.2 (36.85) | 0.408 ^(2)^ | 18 | 71.7 (39.15) | 61 (39.51) | 0.737 | 7 |
| **Fibrinogen**, mg/dL | 315.9 (45.15) | 708.9 (1139.10) | 0.115 ^(2)^ | 18 | 380 (37.99) | 204.7 (137.18) | 0.026 (2) | 7 |
| **Creatinine**, mg/dL | 0.8 (0.20) | 0.9 (0.17) | 0.976 | 51 | 0.8 (0.16) | 0.8 (0.16) | 0.946 | 37 |
| **Glomerular filtration,** ml/min/1.73 m^2 | 64.6 (7.95) | 65.9 (12.53) | 0.468 ^(2)^ | 51 | 64.3 (9.36) | 64.2 (10.08) | 0.464 (2) | 37 |
| **Glucose**, mg/dL | 96.8 (12.72) | 95 (17.61) | 0.149 ^(2)^ | 51 | 98.6 (10.76) | 95.2 (11.58) | 0.358 | 38 |
| **Uric acid**, µmol\L | 5.6 (1.12) | 5.2 (1.27) | 0.268 | 51 | 5.6 (0.96) | 5.1 (1.30) | 0.209 | 37 |
| **Leucocytes**, 10/µL | 589.5 (1648.75) | 6.9 (2.88) | 0.199 ^(2)^ | 18 | 6.9 (2.00) | 6 (1.13) | 0.497 | 7 |
| **Neutrophils**, 10/µL | 350.9 (980.70) | 4.2 (2.30) | 0.153 ^(2)^ | 18 | 4.5 (1.65) | 3 (1.80) | 0.305 | 7 |
| **Eosinophils**, 10/µL | 23.5 (65.97) | 0.1 (0.07) | **0.013** ^(2)^ | 18 | 0.2 (0.15) | 0.2 (0.13) | 0.186 (2) | 7 |
| **Basophils**, 10/µL | 4.7 (13.18) | 0.1 (0.05) | 0.113 ^(2)^ | 18 | 0 (0.02) | 0 (0.02) | 0.177 (2) | 7 |
| **Monocytes**, 10/µL | 31.2 (87.18) | 0.5 (0.22) | 0.429 ^(2)^ | 18 | 0.4 (0.02) | 0.4 (0.16) | 0.959 | 7 |
| **Lymphocytes**, 10/µL | 179 (501.05) | 2 (0.45) | 0.5 ^(2)^ | 18 | 1.7 (0.24) | 1.7 (0.25) | 0.977 | 7 |
| **GOT**, U/L | 22.4 (4.30) | 25.2 (8.42) | 0.163 | 51 | 20.1 (4.91) | 25.3 (7.81) | 0.022 | 38 |
| **GPT**, U/L | 21.8 (8.38) | 27.4 (14.28) | 0.084 ^(2)^ | 51 | 20.7 (7.77) | 26.8 (10.26) | 0.052 | 38 |
| **Gamma GT**, U/L | 30.6 (28.39) | 30.6 (25.6) | 0.257 ^(2)^ | 50 | 30.6 (22.94) | 32.4 (29.55) | 0.43 (2) | 38 |
| **Alkaline phosphatase**, U/L | 68 (7.52) | 76.6 (21.08) | 0.29 | 18 | 72.7 (11.93) | 71 (13.44) | 0.872 | 7 |
| **Total bilirubin**, mg/dL | 1 (0.27) | 0.7 (0.19) | **0.036** | 18 | 0.8 (0.27) | 0.7 (0.18) | 0.842 | 7 |
| **Total cholesterol**, mg/dL | 210.8 (38.96) | 205.8 (31.95) | 0.619 | 51 | 229.2 (40.08) | 200.8 (31.36) | 0.019 | 38 |
| **LDL cholesterol**, mg/dL | 135.7 (32.71) | 116.9 (35.75) | 0.061 ^(2)^ | 51 | 146.1 (36.43) | 120.4 (24.80) | 0.015 | 37 |
| **HDL cholesterol**, mg/dL | 55.6 (14.20) | 69.8 (33.05) | 0.085 ^(2)^ | 51 | 61.5 (11.80) | 57.7 (18.88) | 0.474 | 38 |
| **Triglycerides**, mg/dL | 97 (41.53) | 109.8 (53.36) | 0.219 ^(2)^ | 51 | 117.8 (101.34) | 111.9 (60.42) | 0.386 (2) | 37 |
| **Rheumatoid factor**, U/mL | 2.4 (2.83) | 2.1 (2.62) | 0.397 ^(2)^ | 17 | 2.7 (3.06) | 0.8 (1.50) | 0.218 (2) | 7 |
| **Sodium**, mmol/L | 142.1 (2.35) | 141.1 (1.84) | 0.308 | 18 | 140.3 (1.11) | 140.2 (1.25) | 0.938 | 7 |
| **Potassium**, mmol/L | 4.2 (0.37) | 4.4 (0.21) | 0.224 | 18 | 4.1 (0.10) | 4.4 (0.16) | 0.039 | 7 |
| **Calcium**, mg/L | 96.6 (3.88) | 95.3 (2.14) | 0.358 | 18 | 96.7 (1.17) | 95 (2.50) | 0.34 | 7 |
| **Magnesium**, mmol/L | 2.1 (0.09) | 2.1 (0.13) | 0.371 | 18 | 2.1 (0.10) | 2.1 (0.13) | 0.439 | 7 |
| **Chloride**, mmol/L | 105.5 (1.60) | 105.4 (1.26) | 0.337 ^(2)^ | 18 | 103.7 (1.15) | 104.5 (1.29) | 0.419 | 7 |
| **Bicarbonate**, mmol/L | 27.4 (1.69) | 27.9 (1.73) | 0.527 | 18 | 28 (1.73) | 26.3 (2.87) | 0.398 | 7 |

Mean values (SD) are shown. **^(1)^** Student's t-test; **^(2)^** Mann-Whitney U test. GOT: Glutamic Oxaloacetic Transaminase; GPT: Glutamic Pyruvic Transaminase; GT: glutamyl transferase; HDL: High-Density Lipoprotein; LDL: Low-Density lipoprotein; SD: Standard Deviation; SPMs: specialized pro-resolving lipid mediators; TT: Thrombin Time; PTT: Partial Thromboplastin Time.
